# Increased number of structured diabetes education attendance was not associated with the improvement in patient-reported health-related quality of life: results from Patient Empowerment Programme (PEP)

**DOI:** 10.1186/s12955-015-0324-3

**Published:** 2015-08-12

**Authors:** Carlos K.H. Wong, William C.W. Wong, Eric Y.F. Wan, Winnie H.T. Wong, Frank W.K. Chan, Cindy L.K. Lam

**Affiliations:** Department of Family Medicine and Primary Care, The University of Hong Kong, 3/F, Ap Lei Chau Clinic, 161 Ap Lei Chau Main Street, Ap Lei Chau, Hong Kong; Integrated Care Programs, Hospital Authority Head Office, Hong Kong Hospital Authority, Ap Lei Chau, Hong Kong

## Abstract

**Aims:**

To assess the effect of a structured education intervention, Patient Empowerment Programme (PEP) patient-reported health-related quality of life (HRQOL) among type 2 diabetes mellitus (T2DM) patients, and if positive effect is confirmed, to further explore any association between frequency of sessions attendance and HRQOL.

**Methods:**

A total of 298 T2DM patients were recruited when they attended the first session of PEP, between March and September 2010, and were followed over a one-year period from baseline. HRQOL data were assessed using Short Form-12 Health Survey version 2 (SF-12) and Short Form-6 Dimension (SF-6D) at baseline and one-year follow-up. Individuals’ anthropometric and biomedical data were extracted from an administrative database in Hong Kong. Unadjusted and adjusted analyses of linear regression models were performed to examine the impact of PEP session attendance on the change in the HRQOL scores, accounting for the socio-demographic and clinical characteristics at baseline.

**Results:**

Of the 298 eligible patients, 257 (86.2 %) participated in the baseline assessment and 179 (60.1 %) patients completed the follow-up assessment, respectively. Overall, PEP resulted in a significant improvement in SF-12 bodily pain and role emotional subscales and SF-6D utility scores. These positive changes were not associated with the level of participation as shown in both unadjusted and adjusted analyses.

**Conclusions:**

The PEP made significant improvement in bodily pain, role emotional and overall aspects of HRQOL. Higher number of session attendance was not associated with improvement in HRQOL in primary care real-world setting.

**Key Messages**

● Participants with type 2 diabetes mellitus who participated in structured diabetes education programme made significant improvement in bodily pain and role emotional subscales and SF-6D scores.

● There was no association between the number of sessions attended and any aspect of HRQOL.

## Introduction

Diabetes mellitus is a chronic disease that affects not only the physical well-being of patients but also their quality of life [1]. The presence of diabetes-related complications, especially those of greater severity, is associated with worsened quality of life for patients with type 2 diabetes mellitus (T2DM) [2, 3]. There is also evidence to suggest that diabetes patients are prone to develop psychosocial problems, such as anxiety and depression [4, 5]. Improving patients’ health-related quality of life (HRQOL) is therefore one of the main goals of diabetes care as well as patient education [6].

Patient empowerment or self-management interventions have evolved from a didactic approach to one that needs theoretical foundation. This reflects the increasing concern for addressing individuals’ underlying beliefs and attitudes which are considered important for sustaining self-care practices and improving long-term outcomes [7, 8]. In assessing the effectiveness of these interventions in diabetes and other chronic diseases, patient-reported HRQOL is increasingly being used as an outcome measure to evaluate physical, emotional and social well-being which are not captured in conventional clinical outcomes [1].

There is a paucity of high-quality data on the impact of these interventions on the HRQOL of diabetes patients, as the vast majority of related studies measure metabolic control and cardiovascular risk reduction but few included any patient reported outcomes. In a meta-analysis of studies on group-based diabetes education intervention published as in 2008, only three studies included HRQOL as an outcome were identified [9]. Significant improvement was noted in two out of the three studies [10, 11]; one study found improvements in the subscales but not in the overall HRQOL [12]. Due to the small number of studies and their heterogeneous study tools, the authors concluded that there was insufficient evidence regarding the effectiveness of self-management in improving HRQOL.

The more recent randomised controlled trials investigating the impact of diabetes education on HRQOL also yielded mixed results. The DESMOND study conducted in the UK evaluated the effectiveness of a structured group education program in 824 patients with newly diagnosed T2DM [13]. The investigators found no significant differences in any of the scores for six dimensions of HRQOL (two overall scores and four subscale scores for physical, psychological, social, and environmental) between the intervention and control groups at one-year follow-up. Another trial in the US compared group education, individual education and usual care in 623 adult patients with T2DM [14], and showed that at a mean follow-up of 6.8 months, HRQOL improved significantly with individual education, but remained unchanged with group-based education versus usual care. Previous studies mainly explored clinical benefits of diabetes education intervention and the factors associated with the attendance of such intervention [15] but none has investigated the effects of its attendance pattern on the patient-reported outcomes. Cost-effectiveness analyses utilizing preference-based HRQOL data [16, 17] address whether structured diabetes intervention was cost-effective compared with usual care.

The design of Patient Empowerment Programme (PEP), a structured group-based self-management education program for T2DM in primary care setting, and results of its long-term effects on metabolic control, diabetes-related complications and mortality have been described elsewhere [18–21]. The aims of this study were to examine whether the PEP improved HRQOL in patients with T2DM, whether increase in the number of sessions attended led to better HRQOL, and which components (generic versus diabetes-specific component) of the interventions were associated with better HRQOL outcomes.

## Methods

This was a before-and-after study that evaluated the change in HRQOL from baseline to year 1 following PEP intervention. The PEP was launched in 2010 for the purpose of enhancing the quality of chronic disease management in primary care throughout Hong Kong. The aims of the programme were to provide the diabetic patients with a combination of knowledge, skills and heighted self-awareness regarding their own disease conditions in order to improve their self-esteem, overall health outcome and quality of life. In brief, two non-government organizations (NGOs), highly experienced in community medical service and education, were invited to conduct the program in the first year. The programme covered two main aspects (1) generic self-efficacy enhancement and lifestyle modification component and (2) disease-specific knowledge and skills component. Generic component sessions, with a total duration of usually 8–10 hours (equivalent to 4–5 two-hour sessions), cover the importance of self-management and behaviour modification, healthy diet and regular exercise goal setting and problem-solving skills, sharing on self-monitoring experience, stress coping management, psychosocial support and networking, and communications with healthcare professionals. Each generic component was group-based with a maximum of 30 participants, and was delivered by the well-trained allied health care professionals, mainly social workers and dietitians. Each disease-specific component was delivered by experienced nurses through lecture-based learning sessions covering comprehensive information about diabetes, responsibility of self-care management, medications in diabetes control, and contingency management on hypo- and hyper-glycaemia. Total duration of disease-specific sessions is 5 hours, with 2.5 hours per session. There is no limitation on the number of generic component sessions attended within six months from the date of enrolment, but the disease-specific sessions were limited to two sessions for each enrolled patient. The educational resources and curriculum were designed by the clinicians and nurses and were revised and updated regularly in order to tailor the material for the diabetic patients in Hong Kong.

### PEP participants

A total of 298 adult individuals selected by convenience sampling were recruited into this HRQOL study at the time when the patients attended the first session of PEP from Hong Kong East and New Territory East district cluster in Hong Kong, between 1 March 2010 and 30 September 2010. Majority (257, 86.2 %) were T2DM patients, attended either generic or diabetes-specific sessions, and completed the baseline assessment by telephone within one month from the date of recruitment. Respondents were administered with the socio-demographic questions and HRQOL instruments using the Chinese (Hong Kong) version of SF-12v2 at baseline assessment and at 12-month follow-up by telephone. The HRQOL data were linked with the clinical data including anthropometric and laboratory data extracted from a clinical management system database of the Hong Kong Hospital Authority. The integration of HRQOL data with clinical data provided a comprehensive profile of patients’ health, and validated the diagnosis of T2DM with the primary care physician coding *International Classification of Primary Care* (ICPC-2) of T90 Diabetes non-insulin dependent.

We defined the patients as having hypertension and the presence of diabetic complications according to the diagnosis coding system of *International Classification of Diseases, Ninth Edition, Clinical Modification* (ICD-9-CM) and ICPC-2 in Hospital Authority administrative database. An information letter detailed the study aims, and written informed consent was obtained from all PEP participants. Ethics approval of this study was granted by the institutional review board.

### HRQOL measures

The Chinese (Hong Kong) SF-12 Health Survey has been validated [22] and normed [23] on the general Chinese population in Hong Kong, and thus was used to measure generic HRQOL in the same population with diabetes [24]. It measures eight subscales of HRQOL on physical functioning (PF), role physical (RP), bodily pain (BP), general health (GH), vitality (VT), social functioning (SF), role emotional (RE) and mental health (MH) on a subscale with theoretical range from 0 to 100. A higher score indicates better HRQOL, with a norm-based mean of 50 and standard deviation of 10. The eight subscale scores were aggregated based on population-specific weights to calculate two summary scores, the physical (PCS) and mental component summary (MCS) scores.

The SF-6D is a preference-based measure of health [25], with a six-dimensional health state classification (physical health, role limitation, bodily pain, vitality, mental health, and social functioning) that quantifies a patient’s health for each dimension, and a set of preference-based weights obtained from representative samples of general population. By deriving the SF-6D preference-based score, seven out of twelve items from SF-12 has been selected to retain the minimum loss of descriptive information as described by Brazier and colleagues [26]. The SF-12-derived SF-6D preference-based score was responsive to detect HRQOL improvements over time [27], with theoretical plausible range from 0.315 (the worse possible health state) to 1 (full health), according to Chinese Hong Kong population-specific scoring algorithm [28, 29]. The clinically important difference of SF-6D was regarded as a difference of at least 0.051 [30]. Based on available SF-6D preference-based data, the quality-adjusted life-year (QALYs) can be estimated for cost-effectiveness analysis of PEP in economic evaluation.

### Sample size calculation

According to a meta-analysis [9] of three previous studies, effect size for overall HRQOL outcome of 0.31 was reported. Based on an alpha value of 5 % and power of 80 %, a total of 90 patients were needed to detect the change in HRQOL scores from PEP enrolment at baseline to 12-month follow-up by paired *t*-test with moderate effect size of 0.3.

### Data analysis

Descriptive statistics were used to calculate the baseline characteristics of socio-demographic and clinical data in respondent and non-respondent groups. Differences in baseline characteristics between respondent and non-respondent groups were tested using Mann–Whitney *U*-test for continuous variables or Chi-square test for categorical variables.

We assessed the effects of PEP on HRQOL over time with the HRQOL scores by SF-12 and SF-6D measured at baseline and follow-up. Unadjusted analyses on the changes in HRQOL scores at follow-up from baseline were tested using paired *t*-test. Adjusted analyses of the linear regression models were performed to estimate the number of PEP session attendance on the change in HRQOL scores, accounting for the socio-demographic characteristics and clinical data collected at baseline assessment.

All statistical analyses were performed using STATA Version 12.0. All significance tests were two-tailed and those with a p-value less than 0.05 were considered statistically significant.

## Results

Of 298 PEP participants who agreed to join the study, 257 (86.2 %) eligible patients participated in the baseline assessment and 179 (60.1 %) completed the 12-month follow-up assessment. Baseline characteristics of PEP respondents and non-respondents are summarized in Table 1. There were no significant differences in any baseline characteristics between the respondents and non-respondents.

**Table 1:** Baseline Characteristics of Respondents and Non-respondents in PEP Participants

		Total	Respondents	Non-respondents	
Characteristic		(N = 298)	(N = 257)	(N = 41)	P-value*
Socio-demographic					
Age (year, Mean ± SD)		62.0 ± 9.6	61.7 ± 9.6	63.6 ± 9.6	0.250
Sex (n, %)					0.204
	Male	147 (49.3 %)	123 (47.9 %)	24 (58.5 %)	
	Female	151 (50.7 %)	134 (52.1 %)	17 (41.5 %)	
Smoking status (n, %)					0.535
	Non-smoker	223 (76.1 %)	191 (75.5 %)	32 (80.0 %)	
	Ever smoking	70 (23.9 %)	62 (24.5 %)	8 (20.0 %)	
Alcohol status (n, %)					0.821
	non-drinker	208 (71.0 %)	179 (70.8 %)	29 (72.5 %)	
	Ever drinking	85 (29.0 %)	74 (29.2 %)	11 (27.5 %)	
Educational level (n, %)					0.051
	Secondary or below	17 (5.8 %)	12 (4.7 %)	5 (12.5 %)	
	Tertiary or above	276 (94.2 %)	241 (95.3 %)	35 (87.5 %)	
Clinical					
Biometric Data (Mean ± SD)					
	Weight (kg)	65.3 ± 12.1	65.5 ± 12.4	63.6 ± 10.3	0.363
	BMI (kg/m^2^)	25.7 ± 3.7	25.7 ± 3.7	25.1 ± 3.6	0.332
	Waist (cm)	90.4 ± 9.7	90.6 ± 9.8	88.8 ± 9.7	0.297
	HbA1c (%)	7.2 ± 1.2	7.3 ± 1.2	7.1 ± 0.9	0.495
	SBP (mmHg)	135.9 ± 17.0	136.2 ± 17.4	134.1 ± 14.2	0.456
	DBP (mmHg)	77.2 ± 11.2	77.2 ± 11.6	76.8 ± 8.9	0.830
	LDL-C (mmol/L)	2.8 ± 0.8	2.9 ± 0.8	2.8 ± 0.8	0.806
	TC (mmol/L)	4.7 ± 0.9	4.7 ± 0.9	4.8 ± 1.1	0.747
	HDL-C (mmol/L)	1.3 ± 0.3	1.3 ± 0.3	1.3 ± 0.4	0.873
	TG (mmol/L)	1.5 ± 0.9	1.5 ± 0.9	1.8 ± 1.0	0.091
Duration of DM (year, Mean ± SD)		7.3 ± 5.9	7.3 ± 6.0	7.3 ± 5.5	0.981
Diabetic complication (n, %)		11 (3.7 %)	10 (3.9 %)	1 (2.4 %)	0.647
Hypertension (n, %)		238 (79.9 %)	207 (80.5 %)	31 (75.6 %)	0.464
Treatment Modality (n, %)					0.884
	Diet only	31 (10.4 %)	27 (10.5 %)	4 (9.8 %)	
	Oral and/or insulin treated	267 (89.6 %)	230 (89.5 %)	37 (90.2 %)	

Note:

*HbA1c* Haemoglobin A1c, *SBP* Systolic Blood Pressure, *DBP* Diastolic Blood Pressure, *LDL-C* Low Density Lipoprotein – Cholesterol, *BMI* Body Mass Index, *TC* Total Cholesterol, *TG* Triglycerides

*Statistically different (P < 0.05) between PEP and non-PEP groups by independent *t*-test or Chi-square test, where appropriate

Table 2 presents the descriptive statistics of HRQOL from baseline to 12-month assessment. In general, the PEP participants reported significant improvement in bodily pain, role emotional subscale and SF-6D scores at the 12-month from baseline. Statistically significant, but not clinically significant, improvement in SF-6D (0.026 ± 0.124, P = 0.006) was observed. The changes in HRQOL stratified by total number of PEP session attended are shown in Table 3. One-session attendees reported deterioration in all HRQOL subscales except bodily pain and vitality subscale scores, at the 12-month follow-up; whilst positive changes in all HRQOL subscales but general health were observed in frequent attendees (≥7 sessions). Of the 179 remaining patients who attended either generic or diabetes-specific sessions, 156 (87.2 %) attended at least one session and were defined as generic session attendees while there were 150 (83.8 %) diabetes-specific session attendees. At 12-month, the change in mean SF-6D was numerically higher albeit insignificant in attendees of diabetes-specific sessions than non-attendees (0.033 ± 0.120 vs −0.011 ± 0.138, P = 0.077), and the improvement in SF-6D in generic session attendees was not as significant as that of non-attendees (0.017 ± 0.124 vs 0.086 ± 0.109, P = 0.012). There was no change in the mental aspect of HRQOL associated with the number of attendance at the generic sessions (0.9 ± 8.8 vs −0.3 ± 8.5, P = 0.517) and diabetes-specific sessions (0.9 ± 8.5 vs 0.4 ± 9.8, P = 0.781). Diabetes-specific session attendees had significantly better scores in the physical aspect of HRQOL than non-attendees (1.0 ± 8.6 vs −2.7 ± 7.3, P = 0.032) while association between physical aspect of HRQOL and number of generic sessions attended was found to be insignificant (P = 0.073). Across other HRQOL subscales of SF-12, the diabetes-specific session attendees reported higher physical scores (−9.6 ± 24.5 vs 1.8 ± 19.4, P = 0.006) than non-attendees. The generic session attendees had lower bodily pain scores (19.6 ± 31.9 vs 4.1 ± 27.5, P = 0.015) than those who did not attend the generic sessions. However, the HRQOL were not associated with the total number of PEP sessions attended in both the unadjusted analysis and adjusted analysis when fully adjusted for the baseline characteristics.

**Table 2:** Descriptive Statistics of Health-related Quality of Life, and their Changes from PEP Enrollment at baseline to 12-month follow-up

		PEP Enrollment at Baseline	12-month After Baseline	Change over Time (n = 179)		
HRQOL				Mean	95%CI	P-value*
SF-12v2						
	PF	87.3 ± 22.9	88.3 ± 20.1	1.2 ± 21.5	(−1.9, 4.4)	0.440
	RP	89.5 ± 20.1	89.5 ± 20.5	−0.1 ± 20.7	(−3.1, 3.0)	0.964
	BP	81.4 ± 28.7	86.0 ± 23.9	6.0 ± 28.5	(1.9, 10.2)	0.005
	GH	40.7 ± 25.0	36.5 ± 23.7	−3.6 ± 29.2	(−7.9, 0.6)	0.095
	VT	71.3 ± 26.5	74.0 ± 17.5	3.7 ± 25.5	(0.0, 7.4)	0.052
	SF	91.3 ± 21.2	92.3 ± 19.3	1.0 ± 22.1	(−2.3, 4.2)	0.560
	RE	88.5 ± 19.1	90.8 ± 16.3	2.7 ± 16.9	(0.2, 5.1)	0.034
	MH	79.9 ± 18.1	81.0 ± 15.9	0.5 ± 17.2	(−2.1, 3.0)	0.706
	PCS	49.1 ± 8.9	49.0 ± 7.7	0.4 ± 8.5	(−0.9, 1.6)	0.548
	MCS	56.4 ± 9.5	57.2 ± 7.4	0.8 ± 8.7	(−0.5, 2.1)	0.229
SF-6D		0.847 ± 0.148	0.869 ± 0.135	0.026 ± 0.124	(0.008, 0.044)	0.006

Note:

PEP = Patient Empowerment Programme; HRQOL = Health-related Quality of Life; PF = Physical Functioning; RP = Role Physical; BP = Bodily Pain; GH = General Health; VT = Vitality; SF = Social Functioning; RE = Role Emotional; MH = Mental Health; PCS = Physical Composite Summary; MCS = Mental Composite Summary

*P-value of testing significance in mean changes using paired *t*-test

**Table 3:** Health-related Quality of Life according to the number of PEP session attendance

		PEP session attendance					
HRQOL		1 (n = 25)	2 (n = 49)	3-6 (n = 41)	≥7 (n = 64)	P-value*	
Change in SF-12v2							
	PF	−3.8 ± 16.9	3.5 ± 21.4	2.3 ± 21.0	0.8 ± 23.6	0.546	
	RP	−7.2 ± 23.2	1.3 ± 15.0	−2.3 ± 17.9	3.3 ± 24.4	0.137	
	BP	8.7 ± 33.1	13.5 ± 27.8	−1.2 ± 22.7	3.9 ± 29.6	0.081	
	GH	−11.7 ± 25.3	0.1 ± 24.8	−5.6 ± 33.8	−2.0 ± 30.4	0.360	
	VT	1.9 ± 27.3	4.0 ± 23.9	1.8 ± 27.3	5.5 ± 25.4	0.881	
	SF	−3.8 ± 32.9	0.0 ± 12.4	−1.7 ± 18.4	5.5 ± 24.6	0.203	
	RE	−1.0 ± 16.6	5.5 ± 14.3	−2.0 ± 15.9	5.1 ± 18.9	0.063	
	MH	−4.0 ± 23.0	3.3 ± 14.4	−1.5 ± 14.1	1.4 ± 18.4	0.294	
	PCS	−1.5 ± 9.2	1.9 ± 7.7	−0.4 ± 7.0	0.4 ± 9.5	0.374	
	MCS	−0.8 ± 10.8	1.3 ± 6.9	−0.9 ± 7.7	2.1 ± 9.6	0.249	
Change in SF-6D		0.000 ± 0.139	0.064 ± 0.106	0.001 ± 0.110	0.024 ± 0.134	0.059	
		Generic session attendance		DM-specific session attendance		P-value**	
		Non-attendees	Attendees	Non-attendees	Attendees		DM-specific
HRQOL		0 (n = 23)	≥1 (n = 156)	0 (n = 29)	≥1 (n = 150)	Generic	
Change in SF-12v2							
	PF	4.3 ± 19.4	0.8 ± 21.8	−5.0 ± 15.3	2.5 ± 22.4	0.458	0.083
	RP	3.3 ± 10.1	−0.5 ± 21.8	−9.6 ± 24.5	1.8 ± 19.4	0.411	0.006
	BP	19.6 ± 31.9	4.1 ± 27.5	5.0 ± 26.6	6.3 ± 29.0	0.015	0.827
	GH	0.4 ± 17.5	−4.2 ± 30.5	−7.7 ± 27.2	−2.8 ± 29.5	0.476	0.408
	VT	3.3 ± 23.0	3.8 ± 26.0	−1.7 ± 26.2	4.8 ± 25.4	0.929	0.208
	SF	−4.3 ± 9.7	1.7 ± 23.3	−3.3 ± 31.3	1.8 ± 19.9	0.220	0.247
	RE	2.7 ± 10.6	2.7 ± 17.6	−2.1 ± 19.7	3.6 ± 16.2	0.987	0.093
	MH	2.7 ± 17.7	0.2 ± 17.2	1.3 ± 22.2	0.3 ± 16.2	0.508	0.784
	PCS	3.3 ± 7.3	−0.1 ± 8.6	−2.7 ± 7.3	1.0 ± 8.6	0.073	0.032
	MCS	−0.3 ± 8.5	0.9 ± 8.8	0.4 ± 9.8	0.9 ± 8.5	0.517	0.781
Change in SF-6D		0.086 ± 0.109	0.017 ± 0.124	−0.011 ± 0.138	0.033 ± 0.120	0.012	0.077

Note:

*PEP* Patient Empowerment Programme, *HRQOL* Health-related Quality of Life, *PF* Physical Functioning, *RP* Role Physical, *BP* Bodily Pain, *GH* General Health, *VT* Vitality, *SF* Social Functioning, *RE* Role Emotional, *MH* Mental Health, *PCS* Physical Composite Summary, *MCS* Mental Composite Summary

*P-value of testing significance using one-way ANOVA

**P-value of testing significance between non-attendees and attendees using paired *t*-test

## Discussions

In this study we evaluated the effect on HRQOL at one-year of the PEP, delivered in a primary care setting as structured group-based diabetes education intervention by NGOs.

### HRQOL outcomes after PEP

Although there were no significant changes in most of the HRQOL subscales at the 12-month follow up, subscales of bodily pain and role emotional were significantly improved among the PEP participants. This is inconsistent with findings from major randomized controlled trials assessing the one-year effect of group-based structured self-management education intervention on patient-reported outcomes [12, 31, 32]. In a US study, the physical aspect of HRQOL was also found to be unchanged at about seven months after enrollment of diabetes group education while the mental aspect of HRQOL was worsened [14]. The Expert Patient Education (X-PERT) program for group-based self-management intervention for T2DM showed a positive effect of the intervention on the diabetes-specific HRQOL [12] whilst the physical functioning of HRQOL sustained improvements after 12 months [31]. In the ongoing DESMOND intervention delivered in primary care setting [33] and the ROMEO intervention delivered in second care clinics [34], HRQOL maintained and continued to improve beyond the first year.

### Associations of PEP sessions attended with HRQOL outcomes

One of the priori hypotheses was that frequent PEP session attendees were beneficial from the greater level of HRQOL. In general, intensive interventions with more training sessions and a longer duration yielded greater improvement for knowledge and metabolic control, but this did not necessarily apply to self-management behaviours [35]. It is important to highlight that PEP reflected real world situations, different from clinical trials unlike the DESMOND [13, 33] and X-PERT [12]. Based on conceptual framework for diabetic patients proposed by Rubin and Peyrot [1], the HRQOL is affected by not only clinical factors but also modifiable psychosocial factors such as health beliefs, social support, and coping strategies. PEP participants may have coped with diseases more actively, and a greater degree of health beliefs and social support, thereby leading to HRQOL improvement after 12-month. However, increased attendances of two education components, regardless of generic and diabetes-specific components, were not associated with better HRQOL with respect to the physical and mental aspects of SF-12. On the contrary, the patients attended more than five sessions of generic sessions were associated with lower SF-6D scores. As each participant is expected to attend 4–5 sessions of generic conponent, this implied that in generic sessions excessive (>5) attendance of the programme appeared to have a negative impact on SF-6D. Findings of current study allowed the translation of SF-6D preference-based score to QALYs for the subsequent use in economic evaluation. Future studies on the cost-effectiveness analysis of PEP should impose the quality adjustment for PEP participants with respect to the number of sessions attended.

The negative findings were thought to be in part attributable to the poor associations of knowledge and information transferred with the behaviour modifications [8]. Even when the PEP sessions were voluntary, over-exposure of generic information learnt by participants could be somewhat counter-productive and fear-inducing [8] leading to the poor compliance of behavior modifications and negative impact on patient-reported outcomes. Furthermore, benefits of improved clinical outcomes from the PEP intervention were not necessarily implied to the positive impact on patient-reported outcomes. The insignificant change in HRQOL following the education intervention is often regarded as ‘positive’ [7], in part due to the increased feelings of stress and burden.

By using the PEP as an example, increasing the sessions attended was not associated with improved HRQOL in participating structured diabetes education intervention. Other than education session attendance, strengthening other intervention components such as incorporating regular reinforcement, appropriate duration of intervention, delivery of booster sessions, peer support or health educator support, may provide alternative ways of delivering effective diabetes education interventions [35–37]. Further research is needed to determine the important components that should be included in the patient empowerment paradigm [35], and to tailor structured education interventions to those receiving the greatest benefits and those in need.

### Limitations

Limitations of this study should be noted. First, there may be concerns of generalizability and response bias with the small proportion of PEP participants included in the study. For instance, a higher proportion of female participants were found in respondent group when compared non-respondent group. Moreover, the negativity bias may be demonstrated in non-respondents whom did not respond at 12-month follow-up, leading to potential differences between respondents and non-respondents in the outcomes being measured. However, baseline characteristics between respondent and non-respondents showed no significant differences. Secondly, the nature of before-and-after study was unlikely to compare with patients without participation of PEP as control group. We cannot rule out possibility that observed changes in HRQOL may be influenced by general trends in routine clinical practice or other unadjusted clinical factors such as onset of diabetic complications. However, our previous studies [19, 20] reported that there were low annual incidence rates of developing diabetic complications in PEP group, supporting that the changes in HRQOL scores were mainly due to the PEP participation.

## Conclusions

There were no associations between the number of sessions attended and any aspect of HRQOL. Based on the changes in HRQOL outcomes, the increase in the sessions attended was not associated with better HRQOL in participants attending structured diabetes education intervention in real-world setting. Understanding such associations can enhance our ability to make structural diabetes education intervention more effective and cost-effective by setting out the ceiling of sessions attended. Nevertheless, the impact on clinical outcomes should also take into consideration for the recommendation to the threshold for the optimal number of PEP session to be attended.
